# Effectiveness of community-based and community-led interventions to improve the psychosocial well-being of those affected by skin-NTDs: A systematic review

**DOI:** 10.1371/journal.pntd.0013997

**Published:** 2026-02-17

**Authors:** Isabelle Ford, Matthew Willis, Anil Fastenau, Gonnie Klabbers

**Affiliations:** 1 Department of Health, Ethics and Society, Faculty of Health, Medicine and Life Sciences, Maastricht University, Maastricht, Netherlands; 2 Department of Global Health, Institute of Public Health and Nursing Research, University of Bremen, Bremen, Germany; 3 Marie Adelaide Leprosy Centre (MALC), Karachi, Pakistan; 4 German Leprosy and TB Centre (DAHW), Wurzburg, Germany; KEMRI-Wellcome Trust Research Programme: Centre for Geographic Medicine Research Coast, KENYA

## Abstract

**Introduction:**

Skin-related neglected tropical diseases (skin-NTDs) are a subgroup of neglected tropical diseases (NTDs) that present with manifestations of the skin, often leading to stigmatization and discrimination of those affected. Community models may be a promising feature in improving the mental health of those affected by skin-NTDs by providing mental health care or resources in areas without formal mental health services. This review aimed to identify existing community-based interventions for improving the psychosocial well-being of individuals affected by skin-NTDs, as well as to evaluate their effectiveness. Sub-questions focused on identifying which interventions are also community-led and evaluating cost-effectiveness.

**Methodology/Principle findings:**

This study is a systematic review, and the protocol was registered with PROSPERO (CRD420251015152). The interventions were evaluated according to an integrated framework and if provided, the cost-effectiveness of the interventions was also assessed. 16 papers were included in this review, and interventions were grouped as either: (I) Group-based, (II) Counselling, (III) Basic Psychological Support, (IV) Management Programs, or (V) Mixed interventions. Results indicated that community-based interventions were effective in reducing internalized stigma and improving the well-being of persons affected by skin-NTDs. Results emphasized how community leadership of interventions is a foundation for its sustainability. There was sparse information on the cost of interventions.

**Conclusions/Significance:**

The findings emphasize that community leadership and participatory methods are a promising method for research in this field and future research should aim to follow this. While only a few interventions addressed multiple skin-NTDs, there is potential for integration of these interventions, both in addressing multiple diseases and for integration within local health structures. The findings highlight that effective interventions exist to support communities without formal mental health resources to improve the psychosocial well-being of those affected by skin-NTDs.

## Author summary

Skin-NTDs are a subgroup of neglected tropical diseases (NTDs) that manifest on the skin, often resulting in disfigurements and chronic disability. Due to this, persons affected by skin-NTDs are often subject to hostility, discrimination, and social exclusion. This systematic review explores how communities attempt to intervene in the negative psychosocial effects that persons with skin-NTDs often face. Our review included 16 papers. The papers included interventions in 5 categories: Group-based, counselling, basic psychological support, management programs, and mixed interventions. Additionally, four of the papers included interventions where the community itself had roles in leading the intervention. Although evidence on cost-effectiveness was limited, the interventions showed encouraging results to improve the psychosocial outcomes for people affected by skin-NTDs. Our findings highlight a gap in the field of NTD research: The need for attention to one’s mental health and well-being, as well as the need for community involvement in such interventions. Community involvement and leadership can help in creating sustainable and effective interventions in resource-constrained settings where such patients are more likely to be neglected.

## Introduction

### Background

The psychosocial well-being of people affected by skin-related neglected tropical diseases (skin-NTDs) is often negatively impacted by the compounding effects of stigma, hostility, chronic disability, and disfigurement [1,2]. Social exclusion and discrimination from the stigmatization of skin-NTDs also contribute to adverse psychological effects on those affected [3]. Efforts in interventions for the control, prevention, and elimination of Neglected Tropical Diseases (NTDs) and skin-NTDs have increased in recent years, but less attention has been given to the negative psychosocial effects of these conditions [4].

NTDs are a group of 20 diseases that occur in both tropical and subtropical climates [5]. The group of diseases shares a further commonality of being linked with poverty and disproportionately burden the poor [5]. While NTDs are primarily found in the Americas, Southeast Asia, and Sub-Saharan Africa, they tend to flourish in environments with less sanitation, clean water, healthcare, and proximity to animals, which compounds their disproportionate effects on poorer communities [5,6]. NTDs collectively cause an estimated 200,000 deaths per year, with 19 million disability-adjusted life years (DALYs) lost annually, encompassing approximately 1% of the global burden of disease, with data indicating a larger prevalence in Central and East Africa, Brazil, and Yemen [7–9]. It is therefore evident that despite the significant global presence of and burden of NTDs, they tend to be neglected by public health initiatives, research, and drug development [2].

There are several reasons for the global neglect of NTDs. NTDs are intrinsically associated with poverty and inequity [8]. Populations affected by NTDs often live in rural areas, slums, and conflict zones, which predisposes those affected to having less priority in public health initiatives [10]. As NTDs are predominantly found in lower-income settings, there have historically been minimal economic incentives for research and treatment funding [8]. Contributing to the inequity of NTDs is the sociocultural stigma that often surrounds these diseases, which prevents people from seeking healthcare and acts as an obstacle to creating global awareness [8]. Furthermore, the need for individual-based treatment and diagnoses as well as the inability to treat NTDs through efforts such as mass drug administration acts as a barrier for large public health initiatives [2]. The higher mortality rate of diseases such as HIV/AIDS and tuberculosis further contributes to NTDs often being overshadowed in the area of public health [8].

Skin-NTDs are a subgroup of at least 10 NTDs that present with manifestations of the skin, such as skin lesions and disfigurement [11]. The World Health Organization (WHO) classifies the following NTDs as skin-NTDs; “Buruli ulcer; chromoblastomycosis and other deep mycoses (including sporotrichosis); cutaneous leishmaniasis; leprosy; lymphatic filariasis; mycetoma; noma; onchocerciasis; post-kala-azar dermal leishmaniasis; scabies and other ectoparasites (including tungiasis); podoconiosis; and yaws” [12]. Many skin-NTDs have commonalities in their physical presentation. Specifically, wounds are a common symptom of Buruli ulcer, cutaneous leishmaniasis, and yaws. Lymphoedema, the swelling of bodily tissue, is also common, presenting in lymphatic filariasis (LF) and leprosy reactions [13,14]. Additionally, skin-NTDs are co-endemic, with persons and communities being affected by multiple skin-NTDs at once, complicating diagnosis and treatment [15]. Further, skin-NTDs share in the stigmatization and discrimination of those affected, which reinforces a cycle of vulnerability, isolation, and poverty [2,16].

Stigma and exclusion of persons suffering from skin-NTDs have significant implications on one’s mental health and overall psychosocial well-being [16]. Stigma itself may be conceptualized according to Link and Phelan [17]. For stigma to exist, people must recognize human differences, and a dominant culture must provide a link between these differences and undesirable characteristics. People are labeled according to these differences, creating distinct groups that leads to discrimination and undesirable outcomes [17]. Societal discrimination and stigma are common for all skin-NTDs and often continue even after the afflicted person has been cured, creating a long-term impact on their interaction with society. Examples of this include effects on partner relationships, with common instances of avoidance, abandonment, and rejection of partners suffering from skin-NTDs. Furthermore, perceived associations between skin-NTDs and sinfulness can subject afflicted persons to hostile actions, unwanted attention, and gossip [1]. Such actions have negative implications on those affected by skin-NTDs and their mental health, which the WHO defines as a “state of mental well-being that enables people to cope with the stresses of life, realize their abilities, learn well and work well, and contribute to their community” [18]. It is evident that the stigmatizing nature of skin-NTDs negatively affects the mental health and well-being of those affected, acting as a barrier for persons to have meaningful lives, relationships with others, and interactions within their communities. This is further demonstrated in recorded instances of persons affected by skin-NTDs being distanced from their communities while also experiencing feelings of shame and even incrimination [19].

In 2023, the WHO held a meeting to discuss skin-NTDs, which focused on addressing these diseases and indicated a gap in attention to the psychosocial aspects of skin-NTDs [1,20]. Specifically, the WHO noted that persons affected by skin-NTDs face physical, emotional, and social problems that, when accounted for, can increase the burden of disease measured in years lived with disability (YLD) [20]. For example, when adding depressive effects, the YLD of lymphatic filariasis is doubled, indicating the significance of the psychosocial effects of skin-NTDs and how they are often underestimated [21]. The recent attention that the WHO has drawn towards combatting NTDs and skin-NTDs has increased programs and research on the group of diseases [20]. Still, many of these programs pay little attention to the psychosocial effects of skin-NTDs.

Further, the literature indicates how community involvement and interventions can be used to improve mental health [22,23]. People suffering from poor mental health, such as many of those affected by skin-NTDs, experience significant barriers to community involvement and engagement that could be used instead to improve one’s mental health and well-being [22]. Community involvement and interventions for those affected by skin-NTDs may contribute to one’s mental health and well-being, especially in areas with limited formal mental health services. Community-based interventions may further provide greater patient empowerment, giving autonomy to historically vulnerable populations. A recent scoping review researched existing community models for addressing skin-NTDs and found that many such models include person-centered approaches that enhance the participation and inclusion of those affected by skin-NTDs [24]. The review further found that only 30% of their studies addressed mental health, indicating that more evidence is required to understand practices related to the mental health and stigma of those affected by skin-NTDs [24]. Community platforms of interventions are promising in their possible improvements for one’s mental health and psychosocial well-being, but more evidence is necessary to understand these interventions, their effectiveness, and further uses.

The objective of this systematic review was to target the knowledge gap regarding the psychosocial effects of skin-NTDs and interventions used in improving the psychosocial well-being of persons-affected. Understanding how local communities respond better to the stigma and discrimination surrounding skin-NTDs and community-based interventions can provide greater insight into improving mental health and the overall well-being of those affected. Expanding the literature on this topic will increase resources and information to help persons affected by skin-NTDs. This systematic review provides information on the effectiveness of psychosocial interventions and contributes to the Sustainable Development Goals (SDGs) identified by the United Nations [25]. Specifically, the United Nations recognizes NTDs under SDG 3, to ensure healthy lives and well-being for all [25,26].

Therefore, this systematic review aimed to increase the literature and information regarding skin-NTDs and the effectiveness of interventions used by communities to improve the psychosocial well-being of those affected. This systematic review primarily examined community-based interventions with a sub-focus on those that were community-led or initiated. It is noted that all community-led interventions are inherently community-based. Further, as it is important to consider the cost of interventions, a secondary aim was to analyze their cost-effectiveness.

### Theoretical considerations

This study utilized an integrated theoretical framework to evaluate the effectiveness of community-based psychosocial interventions for those affected by skin-NTDs [27]. The overall goal of the framework was to comprehensively assess the benefits and harms, resources used, and cost-effectiveness of community-based interventions aimed at improving the psychosocial well-being of those affected by skin-NTDs [27]. The framework was developed by the fig. [27] and primarily used quantitative indicators to determine intervention effectiveness and cost-effectiveness. The present study adapted this to allow for the inclusion of both qualitative and quantitative analyses of health indicators in the community-based interventions.

There are three domains of the framework used to assess benefits and harms of an intervention: *health*, *community well-being*, and *community processes* [27]. The health domain considers the impact of interventions using information of quality of life for individuals affected by skin-NTDs. The community well-being domain involves the impact on the community as a whole, rather than on individual health. The community processes domain further considers how the community and its values are involved in designing, implementing, and participating in an intervention.

The systematic review primarily focused on analyzing the reported effectiveness of community-based interventions according to the framework previously described [27]. Cost-effectiveness was secondarily considered for each intervention if adequate data were provided. When assessed, cost-effectiveness was analyzed qualitatively to determine a given intervention’s long-term benefits, advantages, and disadvantages [27]. This systematic review followed these theoretical guidelines to analyze the effectiveness of community-based interventions for the psychosocial well-being of those affected by skin-NTDs.

## Methodology

### Ethics statement

This study received ethical clearance on 17/03/2024 from the research ethics committee from the Maastricht University Faculty of Health Medicine, and Life Sciences (FHML-REC).

### Design

The study protocol was registered with PROSPERO (CRD420251015152). This systematic review followed PRISMA guidelines and aimed to collate and analyze evidence on the types of community-based psychosocial interventions for persons affected by skin-NTDs as well as the effectiveness and cost-effectiveness of the interventions [28].

### Search strategy

The search string for PubMed, Web of Science Core Collection, Global Health (EBSCO), and Embase (OVID) are shown in Table 1. The MeSH terms for PubMed were determined using six manually chosen sample papers deemed relevant to the study by the reviewers. The search strands successfully returned all six sample papers. The strands were then converted to the Web of Science Core Collection, Global Health (EBSCO), and Embase (OVID) databases.

**Table 1:** Search string.

Database	Search String
PubMed	(“neglected diseases”[MeSH Terms] OR “NTD”[Title/Abstract] OR “skin neglected tropical disease”[Title/Abstract] OR “elephantiasis”[Title/Abstract] OR “filarial lymphoedema”[Title/Abstract] OR “lymphatic filariasis”[Title/Abstract] OR “leprosy”[Title/Abstract] OR “Hansens disease” [Title/Abstract] OR “buruli ulcer”[Title/Abstract] OR “bairnsdale ulcer” [Title/Abstract] OR “onchocerciasis” [Title/Abstract] OR “river blindness” [Title/Abstract] OR “yaws”[Title/Abstract] OR “framboesia”[Title/Abstract] OR “bouba”[Title/Abstract] OR “pian”[Title/Abstract] OR “scabies”[Title/Abstract] OR “mycetoma”[Title/Abstract] OR “leishmaniasis”[Title/Abstract] OR “noma”[Title/Abstract] OR “gangrenous stomatitis”[Title/Abstract] OR “cancrum oris”[Title/Abstract] OR “podoconiosis”[Title/Abstract] OR “non-filarial lymphoedema”[Title/Abstract] OR “chromoblastomycosis”[Title/Abstract] OR “chromomycosis”[Title/Abstract] OR “ectoparasit*”[Title/Abstract] OR “tungiasis”[Title/Abstract] OR “sporotrichosis”[Title/Abstract]) AND (“quality of life”[MeSH Terms] OR “mental health”[Title/Abstract] OR “wellbeing”[Title/Abstract] OR “mental wellbeing” [Title/Abstract] OR “stigma*”[Title/Abstract] OR “social stigma” [Title/Abstract]) AND (“counselling”[Title/Abstract] OR “communit*”[Title/Abstract] OR “social participation”[Title/Abstract])
Web of Science Core Collection	TS = (“neglected diseases” OR “NTD” OR “skin neglected tropical disease” OR “elephantiasis” OR “filarial lymphoedema” OR “lymphatic filariasis” OR “leprosy” OR “Hansens disease” OR “buruli ulcer” OR “bairnsdale ulcer” OR “onchocerciasis” OR “river blindness” OR “yaws” OR “framboesia” OR “bouba” OR “pian” OR “scabies” OR “mycetoma” OR “leishmaniasis” OR “noma” OR “gangrenous stomatitis” OR “cancrum oris” OR “podoconiosis” OR “non-filarial lymphoedema” OR “chromoblastomycosis” OR “chromomycosis” OR “tungiasis” OR “sporotrichosis” OR “ectoparasite*”) AND TS = (“quality of life” OR “mental health” OR “wellbeing” OR “stigma*” OR “*social stigma” OR “mental wellbeing”) AND TS = (“counseling” OR “communit*” OR “social participation”)
Global Health EBSCO	TI ((“neglected diseases” OR “NTD” OR “skin neglected tropical disease” OR “elephantiasis” OR “filarial lymphoedema” OR “lymphatic filariasis” OR “leprosy” OR “Hansens disease” OR “buruli ulcer” OR “bairnsdale ulcer” OR “onchocerciasis” OR “river blindness” OR “yaws” OR “framboesia” OR “bouba” OR “pian” OR “scabies” OR “mycetoma” OR “leishmaniasis” OR “noma” OR “gangrenous stomatitis” OR “cancrum oris” OR “podoconiosis” OR “non-filarial lymphoedema” OR “chromoblastomycosis” OR “chromomycosis” OR “tungiasis” OR “sporotrichosis” OR “ectoparasite*”) AND (“quality of life” OR “mental health” OR “wellbeing” OR “stigma*” OR “*social stigma” OR “mental wellbeing”) AND (“counseling” OR “communit*” OR “social participation”)) OR AB ((“neglected diseases” OR “NTD” OR “skin neglected tropical disease” OR “elephantiasis” OR “filarial lymphoedema” OR “lymphatic filariasis” OR “leprosy” OR “Hansens disease” OR “buruli ulcer” OR “bairnsdale ulcer” OR “onchocerciasis” OR “river blindness” OR “yaws” OR “framboesia” OR “bouba” OR “pian” OR “scabies” OR “mycetoma” OR “leishmaniasis” OR “noma” OR “gangrenous stomatitis” OR “podoconiosis” OR “non-filarial lymphoedema” OR “chromoblastomycosis” OR “chromomycosis” OR “tungiasis” OR “sporotrichosis” OR “ectoparasite*”) AND (“quality of life” OR “mental health” OR “wellbeing” OR “stigma*” OR “*social stigma” OR “mental wellbeing”) AND (“counseling” OR “communit*” OR “social participation”))
Embase (OVID)	((“neglected diseases” or “NTD” or “skin neglected tropical disease” or “elephantiasis” or “filarial lymphoedema” or “lymphatic filariasis” or “leprosy” or “Hansens disease” or “buruli ulcer” or “bairnsdale ulcer” or “onchocerciasis” or “river blindness” or “yaws” or “framboesia” or “bouba” or “pian” or “scabies” or “mycetoma” or “leishmaniasis” or “noma” or “gangrenous stomatitis” or “cancrum oris” or “podoconiosis” or “non-filarial lymphoedema” or “chromoblastomycosis” or “chromomycosis” or “ectoparasit*” or “tungiasis” or “sporotrichosis”).ti,ab,kf. or (“exp neglected disease/”)) and ((“quality of life” or “mental health” or “wellbeing” or “mental wellbeing” or “stigma*” or “social stigma”).ti,ab,kf. or (“exp quality of life/”) or (“exp social stigma/”)) and ((“counselling” or “communit*” or “social participation”).ti,ab,kf)

### Eligibility criteria

Eligibility criteria included the use of full text English papers that were publicly accessible. There were no restrictions on the publication date or geographical location of the papers. To be incorporated in this systematic review, studies must have included community-based interventions for the psychosocial well-being of persons affected by skin-NTDs. Furthermore, PICOS criteria were used to identify the population, intervention, comparison, outcome, and study type of the papers to be included [29].

The *population* included people affected by skin-NTDs that had experienced effects on their mental health and well-being. The *intervention* studied had to be a community-based intervention designed to improve the mental health of the population of interest. The *comparison* involved the effectiveness of the interventions on improving psychosocial well-being as judged by the researchers. The *outcome* was the reported psychosocial well-being of the participants as reported on the individual and community-levels. The *study-type* included original peer-reviewed qualitative, quantitative, and mixed-methods studies.

For the purposes of this study, community-based interventions were defined according to a modified version of McLeroy et al.’s [30] definition. Community-based interventions are those outside the formal health-care sector, but within the setting of the community itself [30]. Community-led interventions are a subtype of community-based interventions that are instigated by or primarily planned by the local community.

### Data collection and management

A systematic literature search was carried out with the search string developed for each database on 31/03/2025. Rayyan software was used to manage the study selection process [31]. After deduplication, at least two authors each reviewed the results of the search to screen for studies. The screening included a review of the study’s title and abstract. At least two authors then independently reviewed the full text of each potentially relevant article before a consensus was reached on which to include in the data extraction process. Conflict on inclusion of articles was resolved through mediation with the senior author.

### Data extraction and analysis

Data extracted from the selected studies were as follows: location, study design and measurements of effects, sample size, setting of the study, population, disease addressed, intervention used, community processes (with regards to the intervention), outcomes, and the social, cultural, and political contexts. If provided, costs of the intervention were also extracted. These data were analyzed using the theoretical framework on the effectiveness of community-based interventions as described above [27].

### Quality assessment

Methodological quality of the included studies was assessed using CASP checklists specific to the study’s design. CASP checklists were also used to screen for risk of bias in accordance with PRISMA guidelines [28,32].

## Results

**Fig 1:**
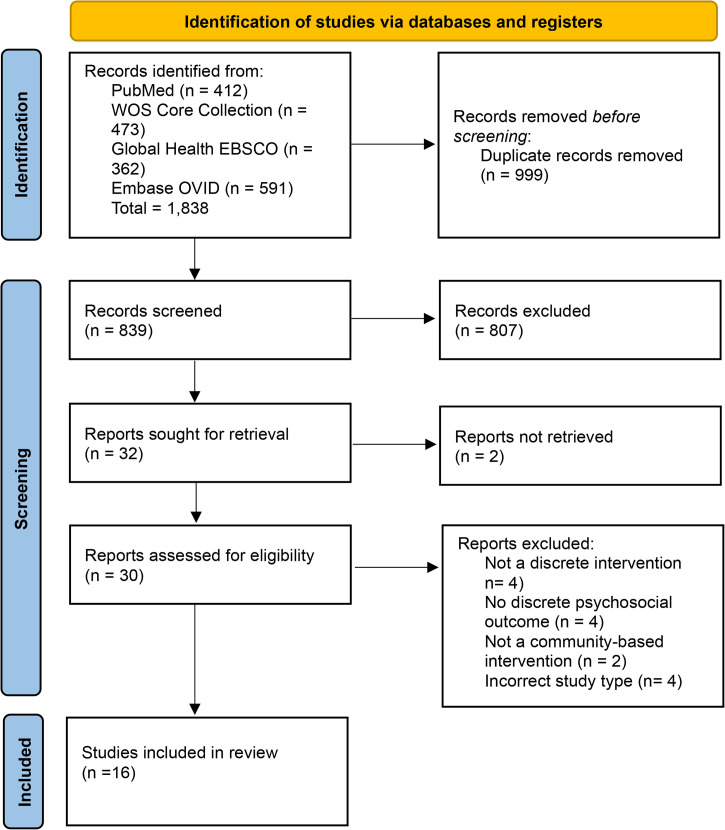
Summary of selection process for papers.

The initial database search yielded 1,838 papers. After deduplication (removing 999 papers), title and abstract screening (removing 807 papers), and full-text screening of 32 papers, 16 papers with nine unique studies were found eligible for inclusion in the analysis (Fig 1). The papers included studies from the Democratic Republic of Congo (DRC) (n = 1), Ethiopia (n = 2), Haiti (n = 1), India (n = 4), Indonesia (n = 4), Nepal (n = 2), Nigeria (n = 1), and Rwanda (n = 1). The studies from India represented the Odisha and Jharkhand states. The papers from Indonesia all reported on interventions from Cirebon District in the West Java province. The papers from Ethiopia reported on an intervention located in the Amhara region.

The papers included are listed with reference, title, study methodology and measurements, country and region, disease addressed, population, intervention, outcomes, community processes, sociopolitical and cultural context, and information regarding cost in S1 Appendix. Methodology used in the studies included qualitative (n = 6), quantitative (n = 6), and mixed methods (n = 4). Furthermore, the skin-NTDs addressed in the studies were leprosy (n = 13), lymphatic filariasis (n = 8), podoconiosis (n = 2), Buruli ulcer (n = 1), and onchocerciasis (n = 1). The interventions found in the review can be separated into five categories: (I) Group-based interventions, (II) Counselling, (III) Basic psychological support, (IV) Management programs, and (V) Mixed interventions. The interventions were assessed using measures related to the mental health and quality of life of individuals affected by skin-NTDs health and relate to the *health*, *community well-being*, and *community processes* domains of the theoretical framework [27].

### Interventions

(I) **Group-based interventions**

Four papers on three unique studies from Nigeria, Nepal, and the DRC assessed group-based interventions. The group-based interventions can be designated as self-help or peer support groups depending on how they are facilitated. Self-help groups were managed by a community team and its group members included people affected by skin-NTDs. Peer support groups were facilitated by persons affected by skin-NTDs, who were trained in providing psychological support. The group-based interventions included activities such as counselling, health literacy and self-care training, and economic development, which occurred in a group setting [33–36].

Disease care was a reported intervention target in both of the self-help groups from Nigeria and the DRC, as well as the peer-supports groups from Nepal [33–36]. Another intervention reported on in the self-help group from Nepal was improving health literacy [35]. The peer support groups from the DRC and Nigeria used participatory methods with the local communities and focused on providing psychosocial support and mental wellness activities, alongside interventions targets such as active case finding, vocational training, and general health [33,36].

Results of the group-based interventions provided information relating to the *health* and *community processes* domains of the theoretical model and specific results are summarized in Table 2 [27,33–36]. All group-based interventions had a significant positive effect on individual *health*, evidenced by decreased internalized stigma, increased resilience and self-esteem. Additionally*,* improvements to *community processes* are demonstrated through the increased ability of persons affected by skin-NTDs to participate in one’s family and community, enhancing their social connections. *Community well-being* was unable to be assessed for all group-based interventions as only two out of the four papers included context on the communities involved, and none of the papers included specific measured relating to *community well-being*.

**Table 2:** Summary of group-based interventions.

Study	Setting	Results
A holistic approach to well-being and neglected tropical diseases: evaluating the impact of community-led support groups in Nigeria using community-based participatory research [33]	Nigeria	Social connections between families and communities were strengthened, participants felt encouraged to openly discuss their feelings and experiences. Participants had improved self-esteem and felt significant reductions in internalized stigma.
From social curse to social cure: A self-help group community intervention for people affected by leprosy in Nepal [34]	Nepal	Negative correlation between one’s access to multiple groups and internalized stigma (*p* = 0.001). Significant indirect relationship of increased self-help group identification on decreased internalized stigma via access to multiple groups (CI [-0.790, -0.197]).
A Resilience Building Collaboration: A Social Identity Empowerment Approach to Trauma Management in Leprosy-Affected Communities [35]	Nepal	Belonging to a self-help group increased access to identity resources (*p* < 0.05), which predicted resilience (*p* < 0.001). Self-help group identification was an important basis for peer-to-peer learning about health (*p* < 0.05 95% CI [.58, 3.40]), and in combination predicted resilience (*p* < 0.01 95% CI [.05,.25]).
Participatory development of a community mental wellbeing support package for people affected by skin neglected tropical diseases in the Kasai province, Democratic Republic of Congo [36]	DRC	Developmental study that showed themes of poverty, depression, and stigma in persons affected by skin-NTDs. Results led to the development of community-led peer support groups.

(II) **Counselling**

Counselling interventions were discreetly studied by three papers, all of which focused on the Cirebon District in Indonesia as a part of the stigma, assessment, and reduction of impact (SARI) project [37–39]. These interventions involved trained counselors helping individuals affected by skin-NTDs to understand their disease, their experiences, and their rights as human beings to improve one’s quality of life and well-being.

Two of the papers studied the “Rights-Based Counselling Module” (RBCM), while the other focused on the role and training of lay and peer counsellors that were used in the RBCM studies [37–39]. RBCM specifically focused on improving affected persons’ knowledge on their human rights, aiming to empower these clients through various forms of individual, group, and family counselling. This counselling was designed to be provided by people with experiences of leprosy or those with connections to people affected by the disease, and required skills such as active listening, showing empathy, and asking effective questions [39].

Specific results are summarized in Table 3; Overall, the studies provide evidence that counselling is an effective intervention for improving the psychosocial well-being of people affected by leprosy. These interventions provided transformative experiences for people affected by leprosy. Patients had powerful realizations of their rights as human beings and became more capable of responding to discrimination and social exclusion. Regarding the *health* domain of the theoretical framework, counselling was effective in decreasing internalized stigma [27]. Counselling further showed positive effects on the *community processes* through increased participation in one’s community and the stimulation of more supportive familial environments. Information regarding *community well-being* was not provided.

**Table 3:** Summary of counselling interventions.

Study	Setting	Results
Lay and Peer Counsellors to reduce leprosy-related stigma--lessons learnt in Cirebon, Indonesia [39]	Indonesia	Clients had powerful realizations of their rights as human beings and felt less alone in their experiences. 56/145 clients did not complete all counselling sessions. Only 9/23 counselors were deemed effective. Not all people affected by leprosy benefited from the experience and may have instead needed an intervention that addressed more than psychosocial needs.
The Impact of a Rights-Based Counselling Intervention to Reduce Stigma in People Affected by Leprosy in Indonesia [38]	Indonesia	Reduction in stigma (SSS) scores from 21.55 to 12.00 (*p* < 0.001). Participation improved, indicated by a reduction in PSS scores from 9.51 to 5.86 (*p* < 0.001). Increased quality of life scores (WHOQOL-BREF), from 80.19 to 86.74 (*p* < 0.001). Clients felt less ashamed of their disease and developed positive self-images. Clients reported feeling more capable to respond to discrimination and stigma, and the counselling developed more supportive family environments. Women had larger reductions in stigma and participation restrictions than men.
Development of a rights-based counselling practice and module to reduce leprosy-related stigma and empower people affected by leprosy in Cirebon District, Indonesia [37]	Indonesia	Identified themes of shame, stigma, and disgust in persons affected. The counselling module was developed to include multiple group settings, as well as reliable knowledge and increased awareness of rights.

(III) **Basic psychological support**

Three papers with two unique studies from India discussed Basic Psychological Support by peers (BPS-N) as an intervention [40–42]. BPS-N is a peer-delivered support program that uses the principles of “Look, Listen, Link” to address psychosocial problems of people affected by skin-NTDs and NTDs in general. These principles are used by peer supporters (PSs) to help identify people in need of support, provide information, address their needs, and connect them to others for social support. Specifically, two of the studies collected pre-intervention, immediate post-intervention, and two months-post intervention data relating to stigma, mental well-being, depressive symptoms, and social participation [40,41]. The third paper built on these results to develop a formal intervention guide [42].

Results of the studies are outlined in Table 4 and relate to the *health* and *community processes* domains of the theoretical framework [27]. Improvements to *health* were evidenced by decreased stigma and improved mental well-being. BPS-N also resulted in reduced depressive symptoms were immediately following the termination of the study. Regarding *community processes*, PSs reported positive experiences, and the intervention helped promote interaction within the community. It is noted, however, that some clients’ expectations were misaligned with the intervention, with some participants requesting material and practical support that fell outside of this intervention’s scope.

**Table 4:** Summary of BPS-N interventions.

Study	Setting	Results
Impact of basic psychological support on stigma and mental well-being of people with disabilities due to leprosy and lymphatic filariasis: a proof-of-concept study [40]	India	Mean level of stigma (SARI) decreased from 30.3 to 24.0 (*p* < 0.001) Mean mental well-being score (WEMWBS) increased from 28.8 to 35.4 (*p* < 0.001). Mean depression scores (PHQ-9) decreased from 12.9 to 8.6 (*p* < 0.001). PSs observed clients opening up more and having an increased ability to meet with their families and communities.
Impact of basic psychological support on stigma and the mental well-being of people with disabilities due to leprosy and lymphatic filariasis: a postintervention evaluation study [41]	India	Reduction of combined number of clients with moderately severe and severe depression from pre-intervention to 2 months post-intervention (*p* = 0.04). Median depression score (PHQ-9) 2 months post intervention (10.5) was significantly higher than immediately post intervention (9.0) (*p* = 0.01). Increasing trend in clients with high mental well-being (*p* < 0.001). Total median stigma score (SARI) decreased from 23.5 post-intervention to 8.0 2 months post-intervention (*p* < 0.001). Some clients reported decreased negative thoughts and increased self-esteem.
A new guide for basic psychological support for persons affected by neglected tropical diseases: A peer support tool suitable for persons with a diagnosis of leprosy and lymphatic filariasis [42]	India	The study developed a guide for BPS-N that focuses on the principles of “Look, Listen, Link”. Guide for PSs to identify people in need of support, approach and listen to them, address their needs, help them cope with their problems, give information, and help connect with social support.

(IV) **Management programs**

Two papers from India and Rwanda studied management programs for specific diseases [43,44]. These interventions were implemented into communities by NGOs and included education and skills-building sessions, allowing affected persons to learn to manage their physical symptoms. Significantly, these programs did not include an intervention to discreetly improve mental well-being. However, these interventions were included due to having effects on mental health and psychosocial well-being in persons affected by skin-NTDs.

The program in India studied the psychosocial effects and social transformation for people with LF-related disabilities using a previously established morbidity management and disability prevention (MMDP) program focused on hygiene, skincare, and lymphedema-reducing activities [43]. The program in Rwanda also studied a previously established program, however, this program focused on podoconiosis prevention and education (PEP) [44]. PEP included interventions for treatment procedures, wearing closed-toed shoes, and leg raising. Both programs were tailored to address specific symptoms and aspects of the individual skin-NTDs LF and podoconiosis, respectively.

The results from both management programs are displayed in Table 5 and provide evidence relating to *health*, *community processes*, and *community well-being* domains of the theoretical framework [27,43,44]. Patients’ experiences in both programs indicate value in the *health* domain, with improvements in participants’ quality of life, and decreased stigma and discrimination. In the PEP intervention, some patients credited improvements to the appearance and odor of their feet to reduced stigma and increased social participation. Additionally, patients had increased knowledge and skills development, along with improved physical symptoms from their disease. Improvements in *community processes* were evidenced in increased community support and positive effects on one’s ability to participate in their community.

**Table 5:** Summary of management programs.

Study	Setting	Results
Experiences of a Community-Based Lymphedema Management Program for Lymphatic Filariasis in Odisha State, India: An Analysis of Focus Group Discussions with Patients, Families, Community Members and Program Volunteers [43]	India	Participants had increased knowledge and skills development, work productivity, and decreased acute episodes. The program resulted in changed perceptions of lymphedema, with improved community acceptance and less obstruction in participation with community events. Community members insisted that discrimination against lymphedema patients was nonexistent.
‘We no longer experience the same pain’: a cross-sectional study assessing the impact of Heart and Sole Africa’s podoconiosis prevention education program [44]	Rwanda	The median QoL score, decreased to by 7 points (*p* < 0.01) 1.6% of participants reported negative changes. There was no significant relationship between QoL and adherence to the program. 98.4% of respondents experienced positive changes in quality of life from PEP (*p* < 0.001). Participants reported a reduction in internalized stigma, and improved sense of community, self-esteem, and social participation.

(V) **Mixed interventions**

Four papers on three unique studies reported on mixed interventions for improving the psychosocial well-being of people affected by skin-NTDs [45–48]. Mixed interventions are defined by combining multiple types of interventions to comprehensively target the effects of skin-NTDs. These interventions often include combinations of management programs, counselling, educational sessions, self-help groups, and community workshops.

Specifically, the study from Indonesia is related to the papers on RBCM and lay and peer counselling from the SARI project reported above and assessed three interventions (counselling, socio-economic development, and community contact), randomly allocating them in pairs to leprosy-affected persons in Cirebon District, Indonesia [45]. The papers from Ethiopia assessed the Excellence in Disability Prevention Integrated across NTDs (EnDPoINT) care package involving education sessions on self-care, counselling sessions, self-help groups, community-level workshops, and training in MMDP. One of these papers was an economic assessment of EnDPoINT and the results are presented elsewhere. The study from Haiti implemented a Chronic Disease Self-Management Program (CDSMP) into peer-led support groups called Hope Clubs that had been previously established in 1998 to provide education, self-care motivation, and opportunities for self-esteem and disease management for women affected by LF [48].

Results of the mixed interventions are summarized in Table 6, excluding the economic assessment. Overall, the mixed interventions resulted in decreased stigma, participation restrictions, and increased quality of life. The specific results varied by intervention, however, as a whole, the mixed interventions appeared to result in improvements across the *health*, *community processes*, and *community well-being* domains [27]. The reduction in stigma and depression, as well as improved psychosocial well-being indicate effectiveness for improving the individual health of persons affected [45,46,48]. Increased community participation, social support, and decreased discrimination in two of the studies provides evidence of larger community benefits as part of the *community processes* domain [45,46]. Additionally, one study provided information on an intervention and its relation to *community well-being* through targeting areas with leprosy-related stigma that lacked existing stigma-reducing interventions [45].

**Table 6:** Summary of mixed interventions.

Study	Setting	Results
Impact of socio-economic development, contact and peer counselling on stigma against persons affected by leprosy in Cirebon, Indonesia – a randomised controlled trial [45]	Indonesia	Mean stigma scores (SSS) decreased from 19.9 to 11.7 (*p* < 0.001) and participation scores (PSS) decreased from 7.6 to 4.9 (*p* < 0.001), indicating a reduction in participation restrictions. Quality of life scores (WHOQOL-BREF) increased from 82.9 to 85.9 (*p* = 0.013). Community stigma scores (EMIC) decreased from 14.4 to 11.0 (*p* < 0.001) and social distance (SDS) reduced from 9.2 to 7.3 (*p* < 0.001). In the control area, stigma scores (SSS) decreased from 15.4 to 9.8 (*p* = 0.009).
Effect of a Community-Based Holistic Care Package on Physical and Psychosocial Outcomes in People with Lower Limb Disorder Caused by Lymphatic Filariasis, Podoconiosis, and Leprosy in Ethiopia: Results from the EnDPoINT Pilot Cohort Study [46]	Ethiopia	All physical measurements improved at 3 months and 12 months after implementation. Quality of life (DLQI) scores improved from 10.9 at baseline to 4.0 at 3 months (*p* < 0.001) and to 3.8 at 12 months (*p* < 0.001). Depressive symptoms (PHQ-9) decreased by an adjusted mean of 5.0 at 3 months (*p* < 0.001) and by 5.1 at 12 months from baseline (*p* < 0.001). Levels of self-reported stigma (adapted ISMI) decreased by a mean of 3.8 at 3 months (*p* < 0.001) and from baseline to 12 months by 5.1 (*p* < 0.001). Mean improvement in discrimination (adapted DISC-12) and social support (OSSS) after 12 months were 3.2 (*p* < 0.001) and 0.6 (*p* = 0.05) respectively.
A pilot study to address the mental health of persons living with lymphatic filariasis in Léogâne, Haiti: Implementing a chronic disease self-management program using a stepped-wedge cluster design [48]	Haiti	No statistically significant differences in SRH, SMCDS, MSPSS, or DLQI between Hope Club and non-Hope Club participants. 8.3% and 52.2% of participants in Arm 1 and Arm 2, respectively, of the Hope Clubs screened positive for depressive symptoms (ZLDSI), which reduced to 34.9% and 34.6% at the midpoint and to 28.7% and 27.6% at the endpoint (*p* = NS). 41.3% power to detect a 10% difference among observation periods assuming a type I error of 0.05.

### Community-led interventions

Of the 16 community-based papers assessed, only four included community-led interventions. The community-led interventions involved three unique studies from the DRC, Nepal, and Nigeria [33–36]. Two of the papers on peer-support groups used participatory research methods, emphasizing co-construction and co-ownership in the development of their interventions [33,36]. The paper from Nigeria specifically highlighted community ownership of the intervention as a significant factor in its sustainability. Similarly, the paper from the DRC noted a significant strength in the co-development of the peer-support groups, however, the impact and sustainability of the intervention cannot fully be known until further studies are conducted.

Of these four studies with community-led interventions, two papers with one distinct study reported on pre-existing self-help groups facilitated by a community team in Nepal. However, no further information on the leadership or how this may have impacted the intervention was provided [34,35]. Another study implemented a new management program intervention into pre-existing community-led support groups [48]. No further information was provided on the leadership of the initial support groups. The majority of other interventions included in this systematic review were implemented from external researchers or NGOs.

### Cost effectiveness

Information regarding the costs of interventions was limited, with only one study providing a significant cost analysis. Six papers described their interventions as being “cost-effective”, “low cost”, or “inexpensive” without providing figures or further information [33,38,42,44,45,48]. These interventions included peer-support groups, lay and peer counselling, management programs, and BPS-N. One paper provided information for a BPS-N intervention, where each peer supporter was paid US $13 per month, with additional stipends for meeting-related travel [40]. Another paper described that lay and peer counsellors were used to lower the costs of the intervention, however, no figures or further information were provided [37]. One paper provided a comprehensive economic assessment of the EnDPoINT care package [47]. The cost of implementation activities was 204,388 ETB (£ 12,263), using the average exchange rate from 2019 and purchasing power parity. The average cost of care supplies and medication was calculated to be 870 ETB (£ 52) per patient, which included custom-made shoes (570 ETB or £34). The study noted that the majority of the costs associated with this intervention were incurred upfront, and when fully adopted, would decrease. No information about the cost in relation to the effectiveness of the interventions was provided in any of the included studies.

### Methodological quality

The methodological quality of the included papers was evaluated using CASP checklists and the results are summarized in S2 Appendix. Papers were assessed using the cohort, cross-sectional, qualitative, randomized controlled trial (RCT), and economic evaluation checklists according to their design. The methodological quality for many of the studies was high. Notably, three of the qualitative studies lacked adequate assessment of the relationship between the researcher and participants [39,40,43]. For four of the qualitative studies, rigorous analysis of the data was unable to be determined [37,39,40,42]. Two of the RCT studies indicated a lack of ‘blinding’ regarding the intervention and loss of participants from the beginning to the end of the studies [38,45]. The cross-sectional papers provided less information or insufficient evidence that the research is valuable without further studies [34,41,44].

## Discussion

**Fig 2:**
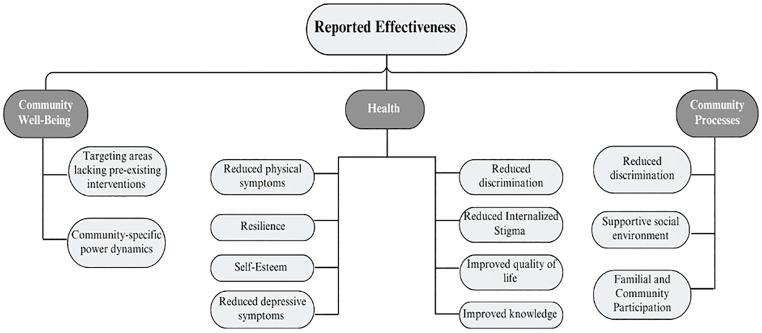
Reported effectiveness of community-based interventions using the theoretical framework by the committee on valuing community-based, non-clinical prevention programs et al. [27].

This review aimed to identify and document the reported effectiveness of community-based interventions to improve the psychosocial well-being of those affected by skin-NTDs. Additional goals were to synthesize information on cost effectiveness and to identify if any of the interventions were not only community-based, but community-led. The current manuscript identified five categories of community-based interventions: (I) Group-Based Interventions, (II) Counselling, (III) BPS-N, (IV) Management programs, and (V) Mixed interventions- targeting themes of stigmatization, social participation, and depression. As a whole, the community-based interventions were able to reduce internalized stigma and depressive symptoms, increase social participation, and provide a knowledge base for skin-NTDs. These results relate specifically to the *health*, *community well-being*, and *community processes* domains of the theoretical framework as outlined in Fig 2.

While the interventions included in this study are all defined as community-based, the majority of studies only assessed individual-level outcomes such as experienced stigma and the ability of persons affected to participate socially. Notably, the majority of the studies failed to incorporate the experiences of the larger community and those that hold stigmatizing perceptions of persons affected by skin-NTDs. This narrow focus may have inadvertently diminished the broader influence of one’s community, especially given that psychosocial well-being is impacted by social environments [49]. This, compounded by the lack of community leadership in the interventions, highlights possible hegemonic power dynamics in the study of neglected diseases. Hegemony involves one dominating group’s worldviews being presented as the norm [50]. In this case, research on skin-NTDs tends to reflect the worldviews and cultural norms of those conducting the research, rather than local communities. This highlights the need for local leadership in NTD research, especially in studies based in affected communities.

Each type of intervention appeared to have positive effects on the mental well-being of persons affected by skin-NTDs. Specifically, group-based, counselling, and BPS-N interventions utilized peers to aid in reducing internalized stigma and the overall improvement of one’s mental well-being [33–42]. The use of peers in these interventions relates to Social Identity Theory, which states that group membership is central to one’s identity and can have large implications on one’s overall well-being [51]. Through the use of peers, these interventions fostered a shared sense of identity and created accepting environments. Similarly, many of these interventions used familial and community support systems. The integration of peer and familial support systems may have also contributed to a sense of group identity, creating a larger system of acceptance for persons affected by skin-NTDs.

However, while counselling and BPS-N interventions proved promising in their overall positive effects on well-being, they may have been limited in their long-term feasibility and sustainability. For example, in the counselling interventions, many lay and peer counsellors were deemed to be ineffective and would require increased training and supervision to ensure long-term sustainability of the program [39]. Additionally, the BPS-N intervention saw a significant increase in depressive symptoms two-months following the termination of the study, indicating that the intervention did not adequately provide skills or resources for persons to individually cope with depression [41]. However, there continued to be a reduction in stigma likely from PSs continuing to see clients, which suggests there is potential for self-sustainability. Furthermore, BPS-N and counselling interventions were limited in their scope and ability to holistically address the needs of clients. While the effects on mental well-being were powerful, they did not address poverty alleviation or access to health care. Focusing discreetly on psychosocial aspects of these diseases neglects the multifaceted nature of skin-NTDs and may inadvertently diminish their physical and financial effects.

This is in contrast to the mixed interventions, which utilized a larger scope to address the needs of persons affected by skin-NTDs [45,46]. Both of the mixed interventions included in this review combined group-based activities with aspects of management programs to address the physical components of skin-NTDs. Interestingly, while management programs did not have a discreet psychosocial component, they had positive effects on mental and physical well-being [43,44]. For instance, treating physical symptoms may have decreased episodes of pain or lead to improvements in physical disfigurements, which can in turn decrease internalized and experienced stigma, improving one’s self-esteem and social participation. While both management programs and mixed interventions provide a broader scope in addressing skin-NTDs, mixed interventions appear to show the most promise in effectively responding to the complex, multifaceted nature of these diseases.

Addressing the multifaceted nature of skin-NTDs aligns with the WHO’s “Ending the neglect to attain the Sustainable Development Goals: A strategic framework for integrated control and management of skin-related neglected tropical diseases” [15]. While the management programs address skin-NTDs on a holistic level, these interventions fall short of fully utilizing the integrated approach advocated for by the WHO. Integration can refer to addressing multiple diseases based on co-endemicity [15]. Of the papers included in this review, only seven included elements of integration by addressing at least two skin-NTDs [33,36,40–42,46,47]. Integration can also refer to embedding interventions within local health care systems [15]. Due to being community-based, these interventions are often designed for specific communities and geographical locations. The context-specific nature of these interventions is promising for future integration within local health care and community structures but was not yet reflected in the majority of the interventions studied. This highlights how designing an integrated intervention is not only dependent upon co-endemicity and shared symptoms of diseases, but also on the contextual nuances specific to each community.

Integration into local health care structures is promising for future interventions and has the potential to yield multiple benefits: it can leverage existing health infrastructures to reduce duplication of efforts, enhance sustainability, and increase acceptance by embedding interventions within community structures [2,15]. This integration into community structures was largely reflected in the community-led interventions assessed that also included components of community leadership. Integration into existing community structures was found in two studies, both of which emphasized participatory research methods and co-ownership of the intervention with the community [33,36]. Participatory methods in these studies allowed for community members to collectively agree on and implement their interventions and were seen as vital in the success and possible long-term sustainability. As demonstrated in one study, community leadership allowed for further adaptations to the intervention, making it both culturally sensitive and contextually appropriate [33].

Eight additional studies included aspects of community consultation and collaboration, which refers to the inclusion of the community in the design of the interventions, through feedback and occasionally the full involvement of community members in the research process [37–43,45,52]. These studies may have involved unequal power dynamics, in which community voices were utilized, but not given the ultimate choice in designing or implementing interventions. This reinforces the hegemonic dynamics discussed previously and can result in misaligned expectations between the researchers and the community or the lack of the intervention to fully address participants’ needs, which was noted in multiple of the papers included in this review [37,39,41].

There was limited evidence regarding the cost of interventions, with six papers providing vague descriptions of being “low cost”, or “inexpensive” and no further figures or data [33,38,42,44,45,48]. Price for the interventions was provided in two papers, with one divulging significant detail in a formal economic assessment [40,47]. This overall lack of detailed cost analysis is surprising, given the significant economic dimension of skin-NTDs, especially in how they disproportionately affect marginalized and impoverished populations [5,53]. Notably, this review has identified a major gap in the literature concerning the overall cost and cost-effectiveness of community-based psychosocial interventions targeting the well-being of persons affected by skin-NTDs. The absence of evidence makes it difficult to assess the feasibility and scalability of such interventions, particularly in resource-limited settings, such as those where skin-NTDs tend to flourish [5,6]. Such information may also be necessary in understanding the potential for integrating interventions into existing healthcare and community structures, as was discussed previously and advocated for by the WHO [15].

However, there are ethical limitations in the use of cost-effectiveness analyses in determining the use or prioritization of health care interventions. For instance, such analyses may result in populations receiving inferior care and a reduced chance at a fair health outcome due to a less suitable intervention being perceived as more cost-effective [54]. Overall, the absence of evidence on cost-effectiveness underscores a broader issue in global health research: while cost-effectiveness is often advocated for, there remains a lack of literature in addressing its potential limitations and implications on the equity and viability of interventions. Further research is needed to both generate cost-effectiveness data and to critically examine the assumptions and ethical implications of applying these frameworks to interventions targeting highly stigmatized and underserved populations.

### Strengths and limitations

This systematic review followed the reporting guidelines as prescribed by PRISMA to collect evidence on community-based interventions for improving the psychosocial well-being of persons affected by skin-NTDs [28]. Adding to the methodological rigor is the inclusion of all skin-NTDs in the search string and the searching of multiple databases. The included studies were also subjected to quality appraisal, which allowed for a comprehensive analysis of the evidence provided. However, this review has a few limitations. Only full text papers written in English were included, possibly causing a misrepresentation of evidence or the exclusion of potentially relevant information. This may explain the lack of papers including the full range of skin-NTDs, as only five skin-NTDs were represented in this review, with 15 of 16 included papers targeting leprosy and LF.

### Recommendations

Community-based interventions to improve the psychosocial well-being of persons affected by skin-NTDs should continue to be prioritized in research and strategies targeting skin-NTDs. Given the complex and multidimensional nature of skin-NTDs, interventions should adopt a multi-component design. For instance, interventions should include a knowledge base both for persons affected and their communities. This knowledge base should focus on providing accurate information on the nature of skin-NTDs and emphasize that physical disfigurements may persist despite the patient being ‘cured’. This aids in addressing misconceptions and reducing stigma rooted in visible skin-related symptoms. Further components should reflect the role of one’s social environment in mental health and well-being by measuring outcomes for both persons affected by skin-NTDs and their communities. In addition, future research should include innovative components in non-traditional psychosocial interventions. Multiple papers in this review provided evidence on the use of the participatory media- particularly, photovoice- as a method for individuals affected by skin-NTDs to visually represent their experiences and reflect on their well-being [33,36].

A shift toward integrated interventions is also strongly recommended. Given the co-endemicity of multiple skin-NTDs in affected regions, designing interventions that address the shared psychosocial consequences across diseases is both equitable and efficient. Such approaches can help ensure that all skin-NTDs are addressed and mitigate possible competition for resource allocation amongst the diseases [2]. Studies should further aim for integration within local health structures and community systems. The use of participatory research methods is encouraged to create contextually and culturally appropriate interventions. Further, these research methods should aim to include elements of community leadership and co-ownership of interventions. Incorporating community voices into intervention design can further ensure that the specific needs of communities are being met and influence the acceptance and success of interventions being implemented.

Future research should consider all skin-NTDs and provide insight into those with lesser-known impacts. This review did not find any interventions targeting persons affected by chromoblastomycosis, mycetoma, noma, and yaws, amongst others. Future research should aim to include these skin-NTDs, as these diseases have commonalities in experiencing stigma and exclusion, which often affects the mental health of those affected [1]. Specifically, research should target persons affected by noma, which was only included in the WHO list of NTDs in 2023 [55]. Despite evidence of noma patients experiencing discrimination and stigma, there remains a gap in the literature on its psychological burden and efforts to combat or alleviate this [55]. Considering the broad range of skin-NTDs in future research is necessary to meet SDG Target 3.3: End the epidemics of AIDS, tuberculosis, malaria and neglected tropical diseases and combat hepatitis, water-borne diseases and other communicable diseases [25,26].

## Conclusion

The results of this review indicate that community-based interventions, evidenced in studies of moderate to high methodological quality, can improve mental health through targeting various aspects of one’s well-being. Effective interventions focused on stigma reduction, education, and community involvement, while often integrating discrete psychosocial components such as counselling. The results further highlighted how community leadership can improve an intervention’s sustainability and potential to have long-term positive effects on one’s well-being. Despite this, many interventions did not include elements of community leadership, which leads to misaligned expectations and the inability of an intervention to fully meet the needs of the population. Many interventions did include aspects of community collaboration and participation, which shows promise for future research inviting communities into larger leadership roles. This review further highlights that despite community involvement, the effects of most interventions were assessed with individual level measurements. This neglects the role that one’s community and social environment have in influencing one’s psychosocial well-being. Furthermore, information on the cost of interventions was sparse. Information on cost could be helpful in determining the sustainability of interventions in impoverished communities affected by skin-NTDs and contribute to the gap in literature on cost-effectiveness. Overall, further research on this topic will help to reduce the psychosocial burden facing persons affected by skin-NTDs and help target the WHO’s SDG 3, to ensure healthy lives and promote well-being [25,26].

## Supporting information

S1 Appendix Results table. (DOCX)

S2 Appendix Methodological checklist. (DOCX)

S3 Appendix PRISMA checklist. (DOCX)
