# Supplementary material for: Effectiveness of community-based and community-led interventions to improve the psychosocial well-being of those affected by skin-NTDs: A systematic review
Source: PLoS Negl Trop Dis. 2026 Feb 17;20(2):e0013997. doi: 10.1371/journal.pntd.0013997 (PMC12923124; doi:10.1371/journal.pntd.0013997)
Supplement: S1 Appendix — (DOCX) [file pntd.0013997.s001.docx]

**S1 Appendix- Data Extraction Table**

| Author and Title | Study methodology and measurements | Country/ region | Disease Addressed | Population | Intervention | Outcomes | Community Processes | Sociocultural and political context | Cost |
| --- | --- | --- | --- | --- | --- | --- | --- | --- | --- |
| Impact of basic psychological support on stigma and mental well-being of people with disabilities due to leprosy and lymphatic filariasis: a proof-of-concept study  Agarwal et al., 2023 | Pre and post intervention design (Quasi-experimental) using mixed methods. Pre-intervention and post-intervention scores using standard scales for stigma (SARI), mental well-being (WEMWBS), depression (PHQ-9), and participation (PSSS). | Bokaro (Jharkhand), India | Leprosy and Lymphatic Filariasis (LF) | 90 people, each participant was a resident of the study area with a leprosy or LF-related disability. 10 peer supporters were used for the focus group discussion. | Basic psychological support for persons affected by NTDs (BPS-N) by peer supporters (PSs) | Quantitative results indicated that the mean level of stigma decreased from 30.3 to 24.0 (p<0.001); mean mental well-being increased from 0% to 13% (p<0.001); and mean depression level decreased from 12.9 to 8.6 (p<0.001). There was no significant change in participation restrictions (p = 0.497). Qualitative results indicated that clients were more open to talk to PSs, with more community participation, and willingness to access healthcare services. | District officials, community leaders, leprosy colonies and village leaders were briefed on the study. | Many leprosy and LF-affected individuals had not received schooling. This made it difficult for the study to recruit peer supporters, specifically female peers. | Peer supporters were paid US $13 per month and were given additional funds for travel and attending monthly meetings. |
| Impact of basic  psychological support on stigma  and the mental  well-being of people with disabilities due to  leprosy and lymphatic filariasis: a postintervention evaluation study  Mol et al., 2023 | Cross-sectional, using qualitative and quantitative methods concurrently. Used standard scales for stigma (SARI), mental well-being (WEMWBS), depression (PHQ-9), and participation (PSSS). | Chas Block, Bokaro District, India | Leprosy and Lymphatic Filariasis (LF) | The quantitative sample aimed to include all clients from Agarwal et. al (2023) pilot study (n=75). Included clients were also at least 16 years of age with leprosy or LF-related disabilities. Clients had both average and low mental well-being using a baseline scale. 62 clients were used for this portion. The qualitative sample consisted of clients (n = 16) and peer supporters (n = 6) selected using purposive and convenience sampling. | Basic psychological support for persons with NTDs (BPS-N) | The median stigma score decreased from 30.0 pre intervention to 23.5 post intervention to 8.0 2 months post intervention (*p*<0.001). Mental well-being and depression scores did not increase significantly from post intervention to 2 months post intervention (p NS and p = 0.01 respectively).  Reduction in clients with moderately severe and severe depression from pre intervention to 2 months post intervention (p = 0.04). The study reported an increase in clients’ health status from receiving information on treatment and care. PSs reported improvements in clients opening up to them. Many clients wanted more support from PSs, requesting help that was outside the responsibilities of the PS. | Use of peer supporters, several of which continued to provide support after the intervention had finished. | No information provided. | No information provided. |
| A new guide for basic psychological support for persons affected by neglected tropical diseases: A peer support tool suitable for persons with a diagnosis of leprosy and lymphatic filariasis  Nayak et al., 2025 | Qualitative and participatory methodology. Steps included consultation, consensus, collaboration, collection of qualitative information, compilation, adaption, designing and printing of a new BPS-N guide. | India | Leprosy and Lymphatic Filariasis (LF) | Participants guided the development of the intervention. These included experts on mental well-being, engagement in the WHO PFA, experience working with leprosy, and other relevant experts. | Basic Psychological Support for persons with NTDs (BPS-N) with Psychological First Aid (PFA) adaptations | The results of the workshop was the BPS-N intervention guide for use by peer supporters. This included guidance on helping vulnerable people, basic psychological support, tips for peer supporters, and how to provide basic psychological support for NTDs among others. | Persons affected by the diseases participated in the virtual meetings. | No information provided. | Referred to as a “cost-effective” solution. |
| Experiences of a Community-Based Lymphedema Management Program for Lymphatic Filariasis in Odisha State, India: An Analysis of Focus Group Discussions with Patients, Families, Community Members and Program Volunteers  Cassidy et al., 2016 | Focus group discussions with thematic analysis of transcripts. 8 sex-aggregated focus groups with patients and 8 sex-aggregated focus groups with family members. 4 mixed-sex focus groups with program volunteers and 4 focus groups (3 sex-aggregated and 1 mixed) with community members. | Odisha State, India | Lymphatic Filariasis (LF) | 74 patients with lymphedema, 74 family members, 28 volunteers, and 35 community members. | Lymphedema management programme | Participants felt they had increased knowledge and skills development, decreased acute episodes, and increased work productivity. Researchers concluded that participation in the program increased feelings of social acceptance and inclusion, with an overall psychosocial improvement | Community members in villages with the lymphedema management program were involved in the focus group discussions. The intervention involved community outreach activities such as street plays and radio spots. | No information provided. | Discussed economic impacts of living with lymphedema as a major theme. No intervention-specific information. |
| A holistic approach to well-being and neglected tropical diseases:  evaluating the impact of community-led support groups in Nigeria using community-based participatory research  Chowdhury et al., 2023 | Community-based participatory research design. 3 phases of research consisting of a scoping review, photovoice methodology, intervention design, implementation of intervention, photovoice with support groups, interviews with researcher and group members, participant observation, and key informant interviews. | Kwara and Kaduna, Nigeria | Skin NTDs in general, including, leprosy, and buruli ulcer | 58 participants across 10 support groups (32 in Kaduna and 26 in Kwara). Participants either had a skin NTD or were caregivers of relatives with skin NTDs. | Integrated community-led peer support groups | Photovoice was thematically analyzed revealing themes of community acceptance and advocacy, capacity strengthening and independence, and improved health knowledge and health outcomes.  Results indicated increased sense of belonging between group members along with widely reported improved self-esteem and reductions in internalized stigma. | Use of affected community members as co-researchers. Co-researchers supported the design and implementation of the intervention. | 2 study sites chosen for the endemicity of NTDs and sociocultural differences. No further context was given in this paper of the study. | Referred to as “low-cost”. |
| Impact of socio-economic development, contact and peer counselling on stigma against persons affected by leprosy in Cirebon, Indonesia – a randomised controlled trial  Dadun et al., 2017 | Randomized control trial. Use of SARI (SSS), PSSS, WHOQOL-BREF, EMIC-CSS, and SDS scales to assess stigma, participation restrictions, quality of life, community stigma, and social distance respectively. | Cirebon District, Indonesia | Leprosy | 62 people affected by leprosy received pilot counselling. 23 lay and peer counsellors were trained and counselled 145 clients, with a total 207 people affected by leprosy receiving the counselling. 110 people affected by leprosy, 8 people with disability and 251 community members received socio-economic development services.  91 ‘contact’ events in 62 villages, where over 4,400 community members attended. | Peer counselling (RBCM), socio-economic development services, and ‘contact’. ‘Contact’ refers to bringing people affected by leprosy into contact with the community. | Each scale indicated significant improvement in the intervention areas (*p*<0.05), with the control sample showing smaller improvements in stigma reduction as well (*p* = 0.009). | Contact interventions were adapted to the specific communities and involved community members. | High level of leprosy-related stigma in the Cirebon District. Prior to this study, there were no reported initiatives to address this problem. | Referred to as “low-cost” and “inexpensive”. |
| The Impact of a Rights-Based Counselling Intervention to Reduce Stigma in People Affected by Leprosy in Indonesia  Lusli et al., 2016 | Mixed methodology: Scales, interviews, focus group discussions, and reflection notes with comparisons between the intervention and control groups. Standard scales used included the SARI Stigma Scale (SSS) to measure stigma, WHOQOL-BREF to measure quality of life, and PSS for participation. | Cirebon District, Indonesia | Leprosy | 23 people were trained as counselors: 10  affected by leprosy, 6 with physical disabilities, 1 with a visual impairment, and 6 with no disability or impairment. 260 people affected by leprosy were counselled: 62 in the pilot phase and 198 in the intervention phase. 207 of the 260 people became counselling clients. | Rights-Based Counselling Module (RBCM) | SSS total reduced from 21.55 to 12.00 (*p*<0.001)  PSS reduced from 9.51 to 5.86 (*p*<0.001)  WHOQOL-BREF increased from 80.19 to 86.74 (*p*<0.001)  At baseline, counselling clients experienced more stigma and participation restrictions, and had poorer QoL.  Control group had a significant reduction in SSS scores, but these were smaller than the differences seen for counselling clients.  QoL decreased significantly for the control group, while increasing significantly for the counselling group. | Stigmatized peoples are trained as counsellors and involved in the lay and peer counselling services. | High levels of leprosy-related stigma in the Cirebon District. | Referred to as “inexpensive”. |
| Development of a rights-based counselling practice and module to reduce leprosy-related stigma and empower people affected by leprosy in Cirebon District, Indonesia  Lusli et al., 2017 | The first phase consisted of a qualitative exploratory study with interviews and focus group discussions. The second phase used a pilot study to test the counselling module. Use of standard scales include SARI stigma scale (SSS), PSS, and WHO Quality of Life. | Cirebon District, Indonesia | Leprosy | Participants in the interviews involved 53 people affected by leprosy or their caregivers. 5 focus group discussions involved people affected by leprosy, family members, and counselling experts. 62 clients and 20 family members were counselled during the pilot study. | Rights-Based Counselling Module (RBCM) | Many people affected by leprosy had misconceptions regarding their health, people often felt shame and low self-esteem due to their diagnosis, and that many indicated that their basic rights (such as access to medical care) were disrespected or violated. This informed the RBCM intervention, which resulted in significant reductions in SSS, PSS, and WHO Quality of Life scores. | Persons affected by leprosy or disability were recruited to assist in the study. The RBCM intervention was further designed for use by lay and peer counsellors. | No information provided. | Use of peer and lay counsellors to lower the cost of the intervention. No further information provided. |
| ‘We no longer  experience the  same pain’: a  cross-sectional  study assessing  the impact of Heart and Sole Africa’s podoconiosis  prevention education program  Gebreselassie et al., 2024 | Quantitative surveys and Qualitative questions. Change in quality of life was measured using questions relating to symptoms, stigmatization, productivity, and abuse. | Musanze and Burera districts of Rwanda | Podoconiosis | 127 participants, with 80 from Burera and 47 from Musanze. Participant criteria included being at least 18 years of age and enrolled at a HASA center before February of 2021. | Heart and Sole Africa (HASA) lymphedema management | Median QoL score decreased from 14 pre-intervention to 7 post-intervention, indicating improvement in QoL (p<0.01).  Qualitatively, several participants indicated a reduction in internalized stigma, with feelings of appreciation and support from peers. Participants indicated improvements in appearance and odor of their feet also improved self-esteem and social participation. | No information provided. | Large amount of the community’s population is involved in subsistence agriculture on a volcanic landscape, with long-term exposure to volcanic soils being a cause for podoconiosis. Approximately one half of residents live below the poverty line. Musanze has 18 health facilities and Burera has 19, with the average walking distance to a health center being approximately 1 hour. | Described as “low-cost”. |
| Participatory development of a community mental wellbeing support package for people affected by skin neglected tropical diseases in the Kasai province, Democratic Republic of Congo  Nganda et al., 2024 | Implementation research study using photovoice and key-informant interviews | Kasai Province, Democratic Republic of the Congo | The intervention does not target specific skin NTDs, but was implemented in an area endemic for leprosy, LF, and onchocerciasis. | 20 persons at least 18 years of age and affected by leprosy, lymphatic filariasis (LF), or onchocerciasis participated in the photovoice methods. 23 key-informants were interviewed, representing NTD programs of the Ministry of Health, health system stakeholders including medical practitioners, nurses, schoolteachers, community leaders, religious leaders, and traditional healers. | Skin NTD mental wellbeing support package | Workshop participants used the photovoice and informant results to establish community-led peer support groups in the support package. Leaders of the support groups were trained to identify peers struggling with their mental wellbeing and to provide basic psychological support. Activities of the support groups will involve counselling, active case finding, self-sufficiency (generation of income) recreation, home visits, and self-care. | Use of participatory research methods to involve the community. Community stakeholders were interviewed and participated in the workshop to develop the intervention. | The intervention is situated in a post-conflict country, with little known information regarding how this affects skin NTDs. | No information provided. |
| A pilot study to address the mental health of persons living with lymphatic filariasis in Léogâne, Haiti: Implementing a chronic disease self-management program using a stepped-wedge cluster design  Sadiq et al., 2024 | Closed-cohort stepped-wedge cluster trial for existing Hope Clubs. Cross-sectional surveys of non-Hope Club LF patients for baseline comparisons. Use of scales for comparison: Self-Rate Health (SRH), Zanmi Lasante Depression System Inventory (ZLDSI), Self-Efficacy for Managing Chronic Disease 6-Item Scale (SMCDS), Multidimensional Scale of Perceived Social Support (MSPSS), and Dermatology Life Quality Index (DLQI). | Léogâne, Haiti | Lymphatic filariasis (LF) | 10 Hope Clubs with a total of 239 patients were asked to participate and 210 consented. 152 Hope Club members completed the study. 74 non-Hope Club patients completed the baseline survey. | Chronic Disease Self-Management Program (CDSMP) integrated into existing Hope Clubs | No statistically significant differences in SRH, SMCDS, MSPSS, or DLQI between Hope Club and non-Hope Club participants. For Hope Club participants, a positive trend in SRH was observed at the midpoint and endpoint (*p*=NS). Additionally, 48.3% and 52.2% of participants in Arm 1 and Arm 2 respectively, of the Hope Clubs screened positive for depressive symptoms, which reduced to 34.9% and 34.6% at the midpoint and to 28.7% and 27.6% at the endpoint (*p*=NS). | Peer-led community support groups called ‘Hope Clubs’ have been used in Léogâne since 1998. Hope Clubs meet monthly help to educate about LF, provide self-care motivation, and enhance self-esteem and disease management. In 2010 the Hope Clubs began inviting men affected by LF. | Widespread geographic distribution of LF in Haiti has made it difficult to scale up MDA programs for its eradication. Ongoing unrest in Haiti delayed the initiation of the intervention by 3 months. | Described as being “cost effective” and designed for use in settings with limited resources. |
| Effect of a Community-Based Holistic Care Package on Physical and Psychosocial Outcomes in People with Lower Limb Disorder Caused by Lymphatic Filariasis, Podoconiosis, and Leprosy in Ethiopia: Results from the EnDPoINT Pilot Cohort Study  Dellar et al., 2022 | Quasi-experimental before-and-after design to compare the physical and psychosocial characteristics between the baseline, 3 months, and 12 months after implementation. Psychosocial characteristics were compared using the Patient Health Questionnaire-9 (PHQ-9), Dermatology Quality of Life Index (DLQI), Discrimination and Stigma Scale (DISC-12), Internationalized Stigma in Mental Illness scale (ISMI), and the Oslo-3 social support scale (OSSS). | Guagusa Shikudad, Ethiopia | Lymphatic Filariasis (LF), Podoconiosis, and Leprosy | The study invited all people living with LF, podoconiosis, and leprosy in the Gusha area and no formal study size calculation was performed. Additionally, participants must have been at least 18 years of age, living in the district for at least 6 months, and able to communicate in the Amharic language. | EnDPoINT care package | DLQI scores improved from 10.9 at baseline to 4.0 at 3mo (*p*<0.001) and increased to 3.8 at 12 months (*p*<0.001). Depressive scores decreased by an adjusted mean of 5.0 at 3mo from baseline (*p*<0.001) and by 5.1 at 12mo from baseline (*p*<0.001). Levels of self-reported stigma decreased by a mean of 3.8 at 3mo from baseline (*p*<0.001) and from baseline to 12mo by 5.1 (*p*<0.001).  The mean improvement in discrimination after 12mo was 3.2 (*p*<0.001)  The mean improvement in social support after 12mo was 0.6 (*p*=0.005). | No information provided. | Low literacy rates of participants, majority living in rural areas. Relative to their community, 42.2% rated their income as low. | No information provided (see following paper) |
| Economic assessment of a community-based care package for people with lower limb disorder caused by lymphatic filariasis, podoconiosis and leprosy in Ethiopia  Hounsome et al., 2020 | Implementation research study: Phase 1 focused on developing a care package for people with lower limb disorder, Phase 2 was a pilot study to test the care package, and Phase 3 aimed to scale-up the care package in Ethiopia. This study focuses on the economic assessment from Phase 2. | Guagusa Shikudad, Ethiopia | Lymphatic Filariasis (LF), Podoconiosis, and Leprosy | Patients with LF, podoconiosis, and leprosy that presented at the Gusha Health Center were invited to participate. Other inclusion criteria included being at least 18 years of age with the ability to hear and communicate and understand the Amharic language. | EnDPoINT care package | Results of the intervention are presented in the previous article. | No information provided. | No information provided. | The cost for implementing the intervention in an area with 30,558 people was 802,655 Ethiopian birr (ETB) or £48,159 (using 2019 exchange rates). The cost to deliver care to 235 participants was 204,388 ETB or £12,263 (870 EBT or £52 per person). The study further notes that many of the costs are incurred at implementation and, therefore, the cost of the intervention would decrease when the package becomes fully adopted. No information is provided on the projected cost of the intervention after its adoption into the system. |
| From social curse to social cure: A self-help group community intervention for people affected by leprosy in Nepal  Jay et al., 2021 | Measured self-help group identification, perceived access to multiple groups as variables. Criterion variables were measured using the SARI scale and the General Health Questionnaire. | Dhanusha, Mahottari, Sarlahi, and Sindhuli Districts of Nepal | Leprosy | Members of 10 pre-existing self-help groups. The final sample included 98 persons from 18-85 years of age. Each participant had a leprosy diagnosis, with 71 being affected by a leprosy-related disability. | Self-help groups | Findings included a positive correlation between self-help group identification and access to multiple groups ( *r*(96) = 0.37, *p*<0.001). Negative correlation between access to multiple groups and internalized stigma (*r*(96) = -0.39, *p* =0.001). The study reported an indirect relationship between increased self-help group identification and decreased internalized stigma via access to multiple groups (*ab* = -0.42, *SE* = 0.15, 95% CI [-0.790, -0.197]). | No information provided. | The Dalit in Nepal are at higher risk for untreated leprosy impairments due to low status and caste position, of which 54% of participants were Dalit. Majority of study participants had no education. Mean family income of participants was 112,030.86 Nepali rupees, indicating high levels of poverty. | No information provided. |
| A Resilience Building Collaboration: A Social Identity Empowerment Approach to Trauma Management in Leprosy-Affected Communities  Jay et al., 2022 | Longitudinal data collection: Time 1 and Time 2 six months later. Time 1 and Time 2 were each two-week windows of data collection. Use of scales ranging from 0 to 4 and the Likert response format. | Dhanusha, Mahottari, Sarlahi, and Sindhuli Districts of Nepal | Leprosy | Members of 10 pre-existing self-help groups. The initial sample included 98 persons affected by leprosy, 71 of which completed the study. | Self-Help Groups | Belonging to the self-help group acted as a basis for peer-to-peer learning about health (*a^1^*: *b* = 1.99, *SE* = .71, *t*(66) = 2.81, *p* < 0.05 95% CI [.58, 3.40])  which in combination predicted resilience (*b*^1^: *b* = .15, *SE* = .05, *t*(66) = 2.97, *p*< 0.01 95% CI [.05, .25]). Belonging to a self-help group is significantly related to access to identity resources across multiple group domains (*a^2^*: *b* = .67, *SE* = .32, *t*(66) = 2.12, *p*<0.05 95% CI [0.04, 1.29]) which in turn predicted resilience (*b^2^*: *b* = .46, *SE* =.11, *t*(66) 4.17, *p*<0.001 95% CI [.24, .68]). | Self-help groups were pre-existing in the community. | See above. | No information provided. |
| Lay and Peer Counsellors to reduce leprosy-related stigma--lessons learnt in Cirebon, Indonesia  Lusli et al., 2015 | Analysis and monitoring of the notes of and group discussions with lay and peer counsellors. | Cirebon District, Indonesia | Leprosy | 198 people affected by leprosy were offered counselling and 145 accepted the offer. 11 lay and 12 peer counsellors provided counselling services. All peer counselors had previously received MDT and were cured from leprosy. | Lay and peer counselling | Notable results included that the initial contact between the counsellors and the client was important for the client to determine if they wanted to partake in counselling. Involving rapport and building a safe space was key for the counsellors, as well as sharing knowledge on leprosy. Necessary skills for counsellors included empathy, asking effective questions, and motivating self-empowerment and self-confidence. Not all counsellors were deemed effective. Only 9/23 counsellors were determined to be effective and only 56/145 clients attended all counselling sessions. | 8 of the peer counselors were research assistants to the SARI project. | People in Cirebon speak different dialects and have absorbed different religious and cultural differences due to its location near the border of Central-Java. | No information provided. |
