# Supplementary material for: Effectiveness of community-based and community-led interventions to improve the psychosocial well-being of those affected by skin-NTDs: A systematic review
Source: PLoS Negl Trop Dis. 2026 Feb 17;20(2):e0013997. doi: 10.1371/journal.pntd.0013997 (PMC12923124; doi:10.1371/journal.pntd.0013997)
Supplement: S2 Appendix — (DOCX) [file pntd.0013997.s002.docx]

**S2 Appendix- Methodological Checklist Summary**

**CASP- Qualitative**

| Study | Q1 | Q2 | Q3 | Q4 | Q5 | Q6 | Q7 | Q8 | Q9 | Q10 |
| --- | --- | --- | --- | --- | --- | --- | --- | --- | --- | --- |
| Impact of basic psychological support on stigma and mental well-being of people with disabilities due to leprosy and lymphatic filariasis: a proof-of-concept study  Agarwal et. al, 2023 | Yes | Yes | Yes | Yes | Yes | No | Yes | Can’t tell | Yes | Can’t tell |
| A new guide for basic psychological support for persons affected by neglected tropical diseases: A peer support tool suitable for persons with a diagnosis of leprosy and lymphatic filariasis  Nayak et. al, 2025 | Yes | Yes | Yes | Yes | Yes | Yes | Yes | Can’t tell | Yes | Yes |
| Experiences of a Community-Based Lymphedema Management Program for Lymphatic Filariasis in Odisha State, India: An Analysis of Focus Group Discussions with Patients, Families, Community Members and Program Volunteers  Cassidy et. al, 2016 | Yes | Yes | Yes | Yes | Yes | No | Yes | Yes | Yes | Yes |
| A holistic approach to well-being and neglected tropical diseases: evaluating the impact of community-led support groups in Nigeria using community-based participatory research  Chowdhury et. al, 2023 | Yes | Yes | Yes | Yes | Yes | Yes | Yes | Yes | Yes | Yes |
| Participatory development of a community mental wellbeing support package for people affected by skin neglected tropical diseases in the Kasai province, Democratic Republic of Congo  Nganda et. al, 2024 | Yes | Yes | Yes | Yes | Yes | Yes | Yes | Yes | Yes | Can’t tell |
| The Impact of a Rights-Based Counselling Intervention to Reduce Stigma in People Affected by Leprosy in Indonesia  Lusli et. al, 2016 | Yes | Yes | Yes | Yes | Yes | Yes | Yes | Yes | Yes | Yes |
| Development of a rights-based counselling practice and module to reduce leprosy-related stigma and empower people affected by leprosy in Cirebon District, Indonesia  Lusli et. al, 2017 | Yes | Yes | Yes | Can’t tell | Yes | Yes | Yes | Can’t tell | Yes | Yes |
| Lay and Peer Counsellors to reduce leprosy-related stigma--lessons learnt in Cirebon, Indonesia  Lusli et. al, 2015 | Yes | Yes | Yes | Yes | Yes | No | Yes | Can’t tell | Yes | Can’t tell |

**Questions Qualitative:**

1. Was there a clear statement of the aims of the research?
2. Is a qualitative methodology appropriate?
3. Was the research design appropriate to address the aims of the research?
4. Was the recruitment strategy appropriate to the aims of the research?
5. Was the data collected in a way that addressed the research issue?
6. Has the relationship between researcher and participants been adequately considered?
7. Have ethical issues been taken into consideration?
8. Was the data analysis sufficiently rigorous?
9. Is there a clear statement of findings?
10. How valuable is the research?

**CASP- RCT**

| Study | Q1 | Q2 | Q3 | Q4 | Q5 | Q6 | Q7 | Q8 | Q9 | Q10 | Q11 |
| --- | --- | --- | --- | --- | --- | --- | --- | --- | --- | --- | --- |
| Impact of socio-economic development, contact and peer counselling on stigma against persons affected by leprosy in Cirebon, Indonesia – a randomised controlled trial  Dadun et. al, 2017 | Yes | Yes | No | No | Yes | Yes | Yes | Yes | Yes | Yes | Can’t tell |
| A pilot study to address the mental health of persons living with lymphatic filariasis in Léogâne, Haiti: Implementing a chronic disease self-management program using a stepped-wedge cluster design  Sadiq et. al, 2024 | Yes | Yes | Yes | Yes | Yes | Yes | Yes | Yes | Yes | Can’t tell | Can’t tell |
| The Impact of a Rights-Based Counselling Intervention to Reduce Stigma in People Affected by Leprosy in Indonesia  Lusli et. al, 2016 | Yes | Yes | No | No | Yes | Yes | Yes | Yes | Yes | Can’t tell | Yes |

**Questions RCT:**

1. Did the study address a clearly formulated research question?
2. Was the assignment of participants to interventions randomised?
3. Were all participants who entered the study accounted for at its conclusion?
4. Were the participants ‘blind’ to intervention they were given? Were the investigators ‘blind’ to the intervention they were giving to participants? Were the people assessing/analysing outcome/s ‘blinded’?
5. Were the study groups similar at the start of the randomised controlled trial?
6. Apart from the experimental intervention, did each study group receive the same level of care (that is, were they treated equally)?
7. Were the effects of intervention reported comprehensively?
8. Was the precision of the estimate of the intervention or treatment effect reported?
9. Do the benefits of the experimental intervention outweigh the harms and costs?
10. Can the results be applied to your local population/in your context?
11. Would the experimental intervention provide greater value to the people in your care than any of the existing interventions?

**CASP- Cross-Sectional**

| Study | Q1 | Q2 | Q3 | Q4 | Q5 | Q6 | Q7 | Q8 | Q9 | Q10 | Q11 |
| --- | --- | --- | --- | --- | --- | --- | --- | --- | --- | --- | --- |
| ‘We no longer experience the same pain’: a cross-sectional study assessing the impact of Heart and Sole Africa’s podoconiosis prevention education program  Gebreselassie et. al, 2024 | Yes | Yes | Yes | Can’t Tell | Can’t Tell | Can’t Tell | Yes | Yes | Yes | Yes | No |
| Impact of basic psychological support on stigma  and the mental well-being of people with disabilities due to leprosy and lymphatic filariasis: a postintervention evaluation study  Mol et. al, 2023 | Yes | Yes | Yes | Can’t Tell | Yes | Can’t Tell | Yes | Yes | Yes | Yes | Can’t Tell |
| From social curse to social cure: A self-help group community intervention for people affected by leprosy in Nepal  Jay et. al, 2021 | Yes | Can’t Tell | Yes | Yes | Yes | Yes | Yes | Yes | Yes | Yes | Can’t Tell |

**Questions Cross-Sectional:**

1. Did the study address a clearly focused issue?
2. Did the authors use an appropriate method to answer their question?
3. Were the subjects recruited in an acceptable way?
4. Were the measures accurately measured to reduce bias?
5. Were the data collected in a way that addressed the research issue?
6. Did the study have enough participants to minimise the play of chance?
7. How are the results presented and what is the main result?
8. Was the data analysis sufficiently rigorous?
9. Is there a clear statement of findings?
10. Can the results be applied to the local population?
11. How valuable is the research?

**CASP- Cohort**

| Study | Q1 | Q2 | Q3 | Q4 | Q5 | Q6 | Q7 | Q8 | Q9 | Q10 | Q11 | Q12 |
| --- | --- | --- | --- | --- | --- | --- | --- | --- | --- | --- | --- | --- |
| Impact of basic psychological support on stigma and mental well-being of people with disabilities due to leprosy and lymphatic filariasis: a proof-of-concept study  Agarwal et. al, 2023 | Yes | Yes | Yes | Yes | No | Can’t tell | Yes | Yes | Can’t tell | Yes | Yes | Can’t tell |
| A Resilience Building Collaboration: A Social Identity Empowerment Approach to Trauma Management in Leprosy-Affected Communities  Jay et. al, 2022 | Yes | Yes | Yes | Yes | Can’t tell | Yes | Yes | Yes | Yes | Yes | Yes | Yes |
| Effect of a Community-Based Holistic Care Package on Physical and Psychosocial Outcomes in People with Lower Limb Disorder Caused by Lymphatic Filariasis, Podoconiosis, and Leprosy in Ethiopia: Results from the EnDPoINT Pilot Cohort Study  Dellar et. al, 2022 | Yes | Can’t tell | Yes | Yes | Yes | No | Yes | Yes | Can’t tell | Yes | Yes | Can’t tell |

**Questions Cohort:**

1. Did the study address a clearly focused issue?
2. Was the cohort recruited in an acceptable way?
3. Was the exposure accurately measured to minimise bias?
4. Was the outcome accurately measured to minimise bias?
5. Have the authors identified all important confounding factors? Have they taken account of the confounding factors in the design and/or analysis?
6. Was the follow up of subjects complete enough? Was the follow up of subjects long enough?
7. What are the results of this study?
8. How precise are the results?
9. Do you believe the results?
10. Can the results be applied to the local population?
11. Do the results of this study fit with other available evidence?
12. What are the implications of this study for practice?

**CASP- Economic Evaluation**

| Study | Q1 | Q2 | Q3 | Q4 | Q5 | Q6 | Q7 | Q8 | Q9 | Q10 | Q11 | Q12 |
| --- | --- | --- | --- | --- | --- | --- | --- | --- | --- | --- | --- | --- |
| Economic assessment of a community-based care package for people with lower limb disorder caused by lymphatic filariasis, podoconiosis and leprosy in Ethiopia  Hounsome et. al, 2020 | Yes | Yes | Yes | Yes | Yes | Yes | Yes | Yes | Yes | Yes | Can’t tell | Can’t tell |

**Questions- Economic**

1. Was a well-defined question posed?
2. Was a comprehensive description of the competing alternatives given?
3. Does the paper provide evidence that the programme would be effective?
4. Were the effects of the intervention identified, measured and valued appropriately?
5. Were all important and relevant resources required, and health outcome costs for each alternative identified, measured in appropriate units and valued credibly?
6. Were costs and consequences adjusted for different times at which they occurred (discounting)?
7. What were the results of the evaluation?
8. Was an incremental analysis of the consequences and cost of alternatives performed?
9. Was an adequate sensitivity analysis performed?
10. Is the programme likely to be equally effective in your context or setting?
11. Are the costs translatable to your setting?
12. Is it worth doing in your setting?
